# Body Dysmorphic Disorder (BDD): Prevalence in the general population of Pakistan and its association with social media usage

**DOI:** 10.1192/j.eurpsy.2023.532

**Published:** 2023-07-19

**Authors:** S. Azeem, A. Liaquat, Z. Huda, B. Mustafa, N. Iqbal, A. S. Ahmad, M. Adil, A. Ellahi, S. M. A. Jahangeer Al’Saani

**Affiliations:** ^1^King Edward Medical University, Lahore; ^2^Dow University of Health Sciences, Karachi; ^3^Shalamar Medical and Dental College, Lahore; ^4^ Jinnah Sindh Medical University; ^5^Community Medicine, Dow University of Health Sciences, Karachi, Pakistan

## Abstract

**Introduction:**

Body Dysmorphic Disorder (BDD) is a psychiatric, obsessive-compulsive disorder characterized by persistent, pre-occupying, intrusive thoughts regarding defects in one’s physical appearance. This leads to potentially harmful behaviors such as constant mirror checking, avoiding socialization, and the need to seek constant validation. The recent increase in social media usage and influence, especially the use of photo-editing apps, has been correlated with a steep decline in body satisfaction due to the perpetuation of unrealistic beauty standards.

**Objectives:**

This study aimed to determine the current prevalence of BDD in the general population of Pakistan and assess the association of BDD with social media usage.

**Methods:**

A descriptive cross-sectional study was conducted from August 2022 to October 2022 on the general population of Pakistan using an online self-administered, anonymous, pretested questionnaire. It contained socio-demographic factors (age, gender, marital status, educational discipline, and household income). Participants were screened for BDD using a pre-tested Body Dysmorphic Disorder Questionnaire (BDDQ) modified to fit the revised DSM-5 criteria. They were further asked about the specifics of the defects they were concerned about, and whether or not they compared their appearance with people online. Characteristics of social media use such as the types of applications used and time spent on them were also asked. Data was analyzed using SPSS v.26.

**Results:**

Out of 779 participants, 5.3% (41) screened positive for BDD. The most repeated behaviors in BDD-positive participants were a comparison of how they looked with other people and checking themselves in the mirror to see how they look. Their most common defect of concern was skin (acne, scars, wrinkles, paleness, redness) followed by the shape or size of the nose, mouth, jaws, or lips. There was a significant association between age and BDD diagnosis (p < 0.001) and between the tendency to compare themselves with people online and BDD diagnosis (p = 0.001). However, no statistically significant differences were found in the BDD-positive and negative groups concerning gender, the number of social media applications used, or time spent on social media.

**Conclusions:**

There is a need to educate the public about the risk of BDD, especially the more susceptible age, and promote safe social networking. Counseling about the harmful effects of social media could be helpful. This is the first study of its kind done on the Pakistani population and one of the few studies that exist on this topic worldwide. Hence, to reach a conclusive decision, there is a dire need to carry out similar investigations with larger sample sizes and on populations that have yet not been studied.

**Disclosure of Interest:**

None Declared

